# In-Vitro Biofilm Formation and Antimicrobial Resistance of *Escherichia coli* in Diabetic and Nondiabetic Patients

**DOI:** 10.1155/2019/1474578

**Published:** 2019-09-19

**Authors:** Sunayana Raya, Ankit Belbase, Laxmi Dhakal, Krishna Govinda Prajapati, Reena Baidya, Nabin kishor Bimali

**Affiliations:** ^1^Department of Microbiology, Goldengate International College, Battisputali, Kathmandu, Nepal; ^2^Department of Pathology, B&B Hospital, Gwarko, Lalitpur, Nepal

## Abstract

**Background:**

Diabetic patients are more susceptible to urinary tract infection compared to nondiabetic patients, *Escherichia coli* being the most common uropathogen causing UTI. Unreasonable and incorrect antibiotic prescription for UTI in these patients may induce the development of antibiotic-resistant urinary pathogens resulting in delayed recovery and longer hospitalization. In addition to these, biofilm forming capacity of the pathogen may worsen the problem. The main aim of this cross-sectional study (conducted from March to September 2015) is to detect the biofilm forming capacity of UTI causing micro-organisms and compare the antibiotic resistance pattern of *Escherichia coli*, the most common cause of UTI, which will help the physician in choosing the best antibiotic.

**Method:**

Total of 1,099 clean-catch mid stream urine (CCMSU) was processed by standard microbiological technique; 182 were from the diabetic group and 917 nondiabetic. Following identification, all isolates were subjected to antibiotic susceptibility testing using modified Kirby-Bauer disc diffusion method. In-vitro biofilm forming capacity of the isolates were detected by Microtitre plate method. The data were analyzed using SPSS software 16.

**Result:**

Urinary tract infection was found to be significantly higher in diabetic patients (42.9%) compared to nondiabetic patients (17.4%) with *Escherichia coli* as the most common uropathogen in both diabetic and nondiabetic groups. Similarly, UTI was more common in elderly population (29.5%). Imipenem, nitrofurantoin and amikacin were found to be the most effective drug for uropathogenic *E. coli *in both diabetic and nondiabetic patients, whereas amoxicillin, ciprofloxacin, and cotrimoxazole were least effective. Of the total bacterial isolates, 43.3% showed positive results for in-vitro biofilm production by the Microtitre plate method. A significantly higher resistance rate was observed among biofilm producing *E. coli* for quinolones, cotrimoxazole, and third generation cephalosporin ceftriaxone. Most of the biofilm producers (79.5%) were found to be MDR (*p*-value 0.015).

**Conclusion:**

Elderly populations with diabetes are at a higher risk of UTI. Higher biofilm production and resistance to in-use antimicrobial agents in this study render its inefficacy for empirical treatment and point out the importance of biofilm screening to ensure the effective management of infection.

## 1. Introduction

Diabetes is a chronic, metabolic disease characterized by increased levels of blood glucose, which on overtime leads to serious damage to heart, kidney blood vessels, nerves, and eyes. The number of people with diabetes has increased from 108 million (1980) to 422 million (2014) worldwide, causing 1.6 million deaths in 2015 [1, 2]. Diabetic patients are more susceptible to infection compared to nondiabetic counterparts, urinary tract infection being the most common bacterial infection encountered in these patients. Susceptibility of the infection in these patients increases with longer duration and greater severity of diabetes. This results in longer hospitalization, delay in recovery, and creates a sustainable burden in medical costs [3].

UTI is considered as the most common infectious disease affecting the socio-economic life of individual and society [4]. Despite the fact that both male and female are susceptible to UTI, women are more prone to UTI compared to male. Fifty percent of women will develop UTI in their life time and 1 in every 3 women requires antibiotics by the age of 24 due to UTI [5, 6]. The main problem associated with UTI is its recurrence and persistence, which is mainly due to the presence of biofilm associated pathogen [7]. Biofilms are the microbial communities that are irreversibly associated with a surface and are enclosed in a self-produced extracellular polymeric matrix [8]. Bacteria within the biofilm behave differently from their planktonic counterparts especially in terms of antibiotics, which causes limitation in conventional antibiotic therapies [9]. It has been found that bacteria inside the biofilm are 10–1000 times more resistant to antimicrobial agents than the planktonic ones [10]. Increased incidence of diabetes worldwide in recent years and higher rates of antibiotic prescription, for UTI may further impose the development of antimicrobial-resistant urinary pathogens [3].

It has been noted that the etiological characteristics of uropathogen and their antibiotic resistance patterns may vary in different geographic locations, and with time [4, 11]. In Nepal, limited published studies have been done to emphasize the antimicrobial resistance of uropathogen in diabetic patients and biofilm forming capacity of uropathogen. The main aim of this study is to determine the antimicrobial resistant uropathogen among diabetic and nondiabetic patients and detect the in-vitro biofilm forming capacity of uropathogen.

## 2. Material and Methods

### 2.1. Study Design and Population

This cross-sectional study was conducted from March 2015 to September 2015 at one of the Tertiary Hospitals, B&B Hospital, located in Kathmandu valley, Nepal in collaboration with Department of Microbiology of Goldengate International College. A total of 1099 patients (diabetic = 182 and nondiabetic = 917) suspected for UTI visiting the hospital during the study period were included in the study. Diabetic and nondiabetic patients were screened on the basis of blood glucose level. Those whose glucose profile cannot be traced or obtained were excluded from the study.

### 2.2. Ethical Consideration

The study was approved by Institute Review Committee (IRC) of the B&B Hospital.

### 2.3. Sample Collection

Clean-catch midstream urine samples (5–10 ml) were obtained from each patient in sterile screw-capped widemouthed container. The containers were well labeled with unique sample number, date, and time of collection. Samples were transferred to microbiology lab and processed within 1 h of collection.

### 2.4. Sample Processing

The samples were processed by standard microbiological procedure [12]. Presence of ≥10^5^ CFU/ml of one and only type of organism was considered significant bacterial growth. Bacterial susceptibility to antimicrobial agents was determined by the modified Kirby-Bauer disc diffusion method on Muller Hilton agar according to CSLI guideline 2014 [13]. Antibiotics used were amikacin (30 *µ*g), amoxicillin (10 *µ*g), ciprofloxacin (5 *µ*g), ceftriaxone (30 *µ*g), cotrimoxazole (1.25/23.75 *µ*g), nitrofurantoin (300 *µ*g), piperacillin/tazobactum (100/10 *µ*g), imipenem (10 *µ*g), and tetracycline (30 *µ*g). Quality control strains of *Escherichia coli *(ATCC 25922) and *Staphylococcus aureus* (ATCC 25923) were used to validate the results of culture and antibiotic susceptibility test. Isolated organisms were classified as MDR and nonMDR on the basis of antimicrobial resistance pattern. Those isolates resistant to three or more than three groups of antimicrobial agents were classified as MDR [14].

In-vitro biofilm forming capacity of *Escherichia coli* was determined by Microtitre plate method as described by Christensen et al. [15]. The isolates were incubated in a 96-well Microtitre plate containing trypticase soya broth and glucose aerobically at 37°C for 18–24 hours. Then the supernatant was discarded and washed with Phosphate Buffer Saline (PBS). The remaining attached bacteria were fixed with 300 *µ*l of ethanol. The OD values of the isolates that coat the wall of the wells were measured by using ELISA reader after staining with crystal violet. Biofilm producers were classified as negative (nonadherent), weak (weakly adherent), and high (strongly adherent) biofilm producers according to the observed OD values.

### 2.5. Statistical Analysis

Data were entered in Microsoft Office Excel and exported to IBMSPSS version 16.0 (SPSS Inc; Chicago, USA). The association between different variables and UTI was determined using chi-square test, frequency distribution, and univariate logistic regression analysis. An odds ratio with 95% confidence interval was considered statistically significant. *p*-value less than 0.05 was considered statistically significant.

## 3. Results

**Figure 1:**
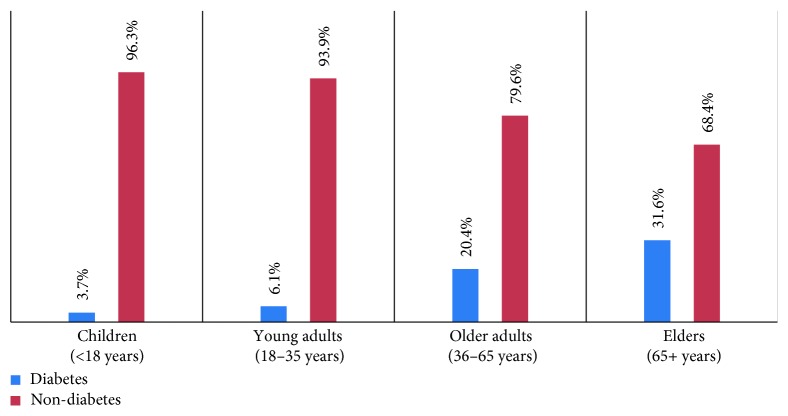
Age distribution of diabetic and nondiabetic patients suspecting UTI.

Total of 1099 urine samples were collected from diabetic patients (182) and nondiabetic patients (917). The mean age of diabetic patient suspecting UTI was 56.5 ± 18.9 and nondiabetic was 41.2 ± 21.2. The incidence of diabetes was higher in older age group i.e., patients over 65 years of age (65,31.6%) compared to other groups (Figure 1).

Out of the total samples, 238 (21.7%) showed significant bacterial growth. The incidence of UTI was found to be higher in female (odds ratio [OR] = 1.518, *p*-value = 0.006, confidence interval [CI] = 1.126–2.045) compared to male. This showed significant association of female with UTI. Similarly, the incidence of UTI was significantly higher in elderly patients (OR = 2.283, *p*-value = 0.005, CI = 1.286–4.055) and diabetic patients (OR = 3.548, *p*-value ≤ 0.001, CI = 2.527–4.983) compared to children and nondiabetic patients, respectively (Table 1).

**Figure 2:**
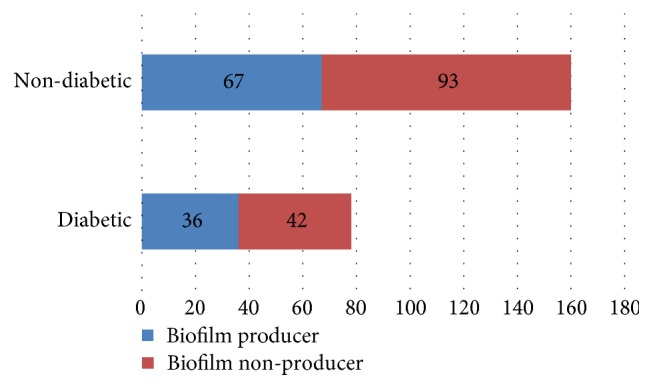
Biofilm production among diabetic and nondiabetic patients.

*Escherichia coli* was the most common cause of urinary tract infection in both diabetic (61, 78.2%) and nondiabetic patients (112, 70.0%) (Table 2). The prevalence of biofilm producing uropathogen was higher in both diabetic (36, 46.2%) and nondiabetic (67, 41.9%) groups (Figure 2).

Of the 238 isolates, 103 (43.3%) were found to be biofilm producers; 92 (38.7%) were weak biofilm producers, and 11 (5.2%) were strong biofilm producers (Table 3).

*E. coli*, the predominant cause of UTI showed higher percentage of resistance to amoxicillin followed by ciprofloxacin in both diabetic and nondiabetic patients. Least resistance rate was observed with imipenem followed by nitrofurantoin, and amikacin. *E. coli* isolated from diabetic patients were found to be more resistant to most of the tested antibiotics compared to nondiabetics. However, no statistically significant relation was found between diabetic and antimicrobial resistance pattern among *E. coli *(Table 4).

No associations of the antibiotic sensitivity pattern were shown among other microorganisms because of the low isolation rate.

Biofilm producing *E. coli* showed comparatively high resistance rate to tested antimicrobial agents than nonbiofilm producing *E. coli *counterparts. The resistance rate of quinolones (CIP), third generation cephalosporin (CTR), and sulphonamide (COT) was statistically higher among biofilm producing *E. coli* (Table 5).

Though MDR *E. coli* was common in both biofilm producers and nonproducers, it was significantly higher in biofilm producer compared to nonbiofilm producing *E. coli* (Table 6).

## 4. Discussion

Diabetes is becoming epidemic and endemic problem in both developing and developed countries with higher prevalence and mortality in elderly population than in adults [16]. This study represented the higher incidence of diabetes in elderly population (31.6%) compared to other age groups. The numbers of studies have evidenced the longer life expectancy in these days as the predisposing factor for diabetes. Decreases in insulin secretion, obesity, and vitamin D deficiency were known to contribute for developing diabetes in elderly population [16].

Out of 1099 urine samples requested for urine culture, 21.7% showed significant bacterial growth. This was in correspondence to the similarly conducted study in Kathmandu valley, Nepal [17, 18]. Diabetic patients have higher odds of acquiring UTI compared to nondiabetic patients. Acharya et al. also showed a higher rate of UTI in diabetic patients (34.5%), but at the lower rate compared to this study [19, 20]. Niveditha et al., in their study, stated diabetes as the most common factor associated with UTI [21]. Nerve damage caused by high blood glucose level affects the ability of the bladder to sense the presence of urine in the bladder thus allowing the urine to stay in the bladder for longer time. The higher glucose level in the urine is known to improve the growth of bacteria in the urine and increased the probability of infection. In addition, the reduced blood circulation (due to prolonged diabetes mellitus) results in abnormalities of the host defense system, increasing the risk of developing infection [20].

Though UTI was found to be prevalent in both sexes and all age groups, it was higher in females (OR = 1.518, *p*-value = 0.006, CI = 1.126–2.045) compared to male. Different other studies also have shown higher rates of UTI in female compared to male [22, 23]. Anatomical proximity of the urethra, sexual activity, and use of spermicidal contraceptives among female have been identified as the predisposing factor for UTI in female [24]. Similarly, the incidence of UTI was higher in elderly population (OR = 2.283, *p*-value = 0.005, CI = 1.286–4.055). Age-associated changes in immune system, exposure to nosocomial pathogens, increasing number of co-morbidity, urinary retention, high post void residual (PVR) urine, and prostatic hypertrophy in men have been postulated as risk factors in the elderly population [25]. On the other hand, increased unhygienic sexual activity in young groups increases the risk of UTI in adults.

*E. coli *was found to be the most common organism associated with UTI in both diabetic and nondiabetic. The ability of uropathogenic *E. coli* to cause infection is associated with expression of diverse virulence factors like fimbriae, siderophores, hemolysin, biofilm formation, etc. [21, 26]. It has been reported that more than 65% of all bacterial infections are related to biofilm production; however, it also depends on the type of the infection and etiology [27]. Previous studies have also highlighted the role of biofilm formation in UTI [28, 29]. Among all the bacterial isolates, 43.4% of the uropathogen were *in-vitro* positive for biofilm formation by the Microtitre plate assay method which corresponds to the study done by Abdagire et al. [30]. The incidence of biofilm production was found to be higher in both diabetic and nondiabetic patients with no statistical relation between them. Though biofilm forming capacity of uropathogen was found to be higher in *in-vitro* condition, it may differ in *in-vivo* condition. Hence, further study regarding the *in-vivo *biofilm forming capacity of uropathogen is necessary in case of relapses and treatment failure.

In this study *E. coli *showed highest percent of resistance to amoxicillin fallowed by ciprofloxacin and cotrimoxazole in both diabetic and nondiabetic patients. A similar study conducted at Tribhuvan University Teaching Hospital also showed amoxicillin as the least effective drug for gram negative bacteria [18]. Another study conducted at Kathmandu University School of Medical Science also presented ampicillin as the least effective drug for uropathogenic *E. coli* [19]. On the other side, imipenem, nitrofurantoin, and amikacin were found to be the most effective drug against uropathogenic *E. coli*. This implies the restricted use of amoxicillin and use of nitrofurantoin for uropathogenic *E. coli*, at least in the study area. Nitrofurantoin is known to be active against a wide range of uropathogen including multidrug resistant gram-negative bacilli and most of the *β*-lactamase-producing strains. However, in some medical conditions use of nitrofurantoin is restricted. *Proteus* spp., *Seratia marcescens,* and *Pseudomonas aeruginosa* are naturally resistant to nitrofurantoin. In these cases, other alternative drugs should be considered [31]. Since, nitrofurantoin and ciprofloxacin are commonly used for the empirical treatment of UTI in Nepal and this study showed high antimicrobial resistance to ciprofloxacin, nitrofurantoin is the only oral drug left. This calls for special attention and further studies regarding the antimicrobial resistance and antimicrobial use.

Antimicrobial resistance is an innate property of bacterial biofilm that may add complications to treatment [32]. The microbial biofilm responds poorly to conventional antibiotic therapy, thus resulting in persistence infections. Antibiotic resistance among biofilm producing *E. coli* was significantly higher for quinolones, trimethoprim-sulfamethoxazole, and third generation cephalosporin ceftriaxone. Increase in antimicrobial resistance within the biofilm arises from multiple factors including reduced bacterial growth rate, local alteration of the micro-environment that may impair activity of antimicrobial agents, EPS, and interbacterial interaction within the biofilm, which promote the spread of drug resistant markers and other virulence factors via gene transfer [8, 33]. Hence the screening of biofilm producing capacity of the isolate prior to antimicrobial treatment is crucial in effective management of the infection.

The isolates resistant to three or more than three classes of antibiotics were considered as MDR [14]. Niranjan and Malini have stated diabetes as the one of the major factors associated with the MDR *E. coli* [34]. Xavier et al. in their study have also reported high levels of MDR isolates in diabetic patients [35]. Nevertheless, our study showed a high degree of MDR *E. coli* in both diabetic (63.9%) and nondiabetic groups (60.7%). The high rates of antibiotic prescription in diabetic patients especially broad-spectrum antibiotics for UTI may be the cause for antibiotic resistant uropathogen [3]. This was in concurrence with the other studies done by Meiland et al., and Chakraborty et al. [36, 37]. The difference in resistance pattern of the bacterial isolates may be due to difference in geographic region, the time of study conducted, and clinical practice. According to Sanchez et al., Alves et al., and Rao et al., biofilm formation increases the resistance profile of the organisms, and the strains that are capable of forming biofilm are mostly MDR phenotypes [32, 38, 39]. A similar report was observed in this study as MDR was about 2 times higher in biofilm forming *E. coli* than nonbiofilm forming *E. coli* (*p*-value = 0.015). Higher prevalence of MDR in biofilm producing strains may be due to transfer of resistant genes to the other organism that initially does not show such resistance [40]. Hence effective strategies for management of biofilm forming *E. coli* are crucial as it may lead to relapses in untreatable UTI.

## 5. Conclusion

Elderly population was found to be the most vulnerable group as diabetes and UTI are more common in this group. Furthermore, high antimicrobial resistant rate may impose a threat causing difficulty in treatment. Continuous surveillance of diabetes and UTI among elder population is necessary to ensure the rational use of antimicrobial agent for empirical and definitive treatment. Quinolones and trimethoprim-sulfamethoxazole, commonly used in treatment of UTI was effective in only less than half number of *E. coli. *Along with this, the resistance rate for quinolone, trimethoprim-sulfamethoxazole and third generation cephalosporin ceftriaxone was significantly higher among biofilm producing isolates and most of the biofilm producing *E. coli* were found to be MDR. This calls for special attention as this may increase the antimicrobial resistance and chronicity of UTI. Further explorations genetically and by in-vivo study would improve our understanding to antimicrobial resistance and provide novel insights into the therapeutics and prevention against biofilm related infection.

## Figures and Tables

**Table 1:** Association of UTI with other variables.

	Growth	No growth	*p*-value	Odds ratio	95% confidence interval
*Sex*					
Male	83 (17.7%)	386 (82.3%)		1	
Female	155 (24.6%)	475 (75.4%)	0.006	1.518	1.126–2.045
*Types of patient*					
Outpatient	163 (21.1%)	610 (78.9%)		1	
Inpatient	75 (23%)	251 (77%)	0.481	1.118	0.820–1.525
*Age group*					
Children (<18 years)	19 (17.4%)	90 (82.6%)		1	
Young adults (18–35 years)	62 (18.8%)	267 (81.2%)	0.742	1.100	0.624–1.939
Older adults (36–65 years)	90 (19.8%)	365 (80.2%)	0.577	1.168	0.677–2.016
Elders (65+ years)	67 (32.5%)	139 (67.5%)	0.005	2.283	1.286–4.055
*Disease*					
Non diabetic	160 (17.4%)	757 (82.6%)		1	
Diabetic	78 (42.9%)	104 (57.1%)	<0.001	3.548	2.527–4.983

**Table 2:** Distribution of uropathogens.

Organism isolated	Diabetic (*n* = 78)	Nondiabetic (*n* = 160)
*Enterobacterales*		
*Escherichia coli*	78.2%	70.0%
*Klebsiella spp.*	9.0%	4.4%
*Enterobacter spp.*	1.3%	2.5%
*Citrobacter spp.*	0.0%	0.6%
*Proteus spp.*	1.3%	3.1%
*Morganella morganii*	0.0%	0.6%
*NonEnterobacterales*		
*Pseudomonas aeruginosa*	3.8%	5.0%
*Acinetobacter spp.*	0.0%	1.3%
*Gram positive*	0.0%	0.0%
Non-Hemolytic streptococci	5.1%	7.5%
Coagulase negative*Staphylococcus*	1.3%	5.0%

**Table 3:** Biofilm production of uropathogens.

Organism isolated	Biofilm		
	Strongly adherent	Weakly adherent	Nonproducer
*Enterobacterales*			
*Escherichia coli*	7	66	100
*Klebsiella spp.*	3	6	5
*Enterobacter spp.*	0	2	3
*Citrobacter spp.*	0	0	1
*Proteus spp.*	0	4	2
*Morganella morgannei*	0	0	1
*NonEnterobacterales*			
*Pseudomonas aeruginosa*	1	5	5
*Acinetobacter spp.*	0	1	1
*Gram Positive*			
Non hemolytic streptococci	0	6	10
Coagulase negative *staphylococcus*	0	2	7
*Total*	**11**	**92**	**135**

**Table 4:** Antimicrobial resistance of *E. coli* isolated from diabetic and nondiabetic patients.

Antimicrobial agents	Diabetic *E. coli* (*n* = 61)		Nondiabetic *E. coli* (*n* = 112)		*P*-value
	Resistance	%	Resistance	%	
*Amikacin*	9	14.8	11	9.8	0.332
*Amoxicillin*	55	90.2	101	90.2	0.998
*Ciprofloxacin*	42	68.9	65	58	0.162
*Ceftriaxone*	33	54.1	64	57.1	0.7
*Cotrimoxazole*	38	62.3	64	57.1	0.51
*Nitrofurantoin*	7	11.5	14	12.5	0.884
*Piperacillin–tazobactum*	23	37.7	35	31.3	0.39
*Imipenem*	5	8.2	8	7.1	0.802
*Tetracycline*	18	29.5	33	29.5	0.995

**Table 5:** Antimicrobial resistance of biofilm producing and nonproducing *E. coli*.

Antimicrobial agents	Biofilm producer *E. coli* (*n* = 73)		Biofilm nonproducer *E. coli* (*n* = 100)		*P*-value
	Frequency	%	Frequency	%		
*Amikacin*	9	12.3%	11	11.0%	0.787
*Amoxicillin*	68	93.2%	88	88.0%	0.261
*Ciprofloxacin*	**55**	**75.3%**	**52**	**52.0%**	**0.002**
*Ceftriaxone*	**51**	**69.9%**	**46**	**46.0%**	**0.002**
*Cotrimoxazole*	**50**	**68.5%**	**52**	**52.0%**	**0.029**
*Nitrofurantoin*	9	12.3%	12	12.0%	0.0948
*Piperacillin–tazobactum*	26	35.6%	32	32.0%	0.619
*Imipenem*	7	9.6%	6	6.0%	0.376
*Tetracycilin*	19	26.0%	32	32.0%	0.395

Bold value indicates the significance difference between the antimicrobial resistance pattern of biofilm producing and non-producing *E. coli* for respective antimicrobial agents.

**Table 6:** MDR profiling of *E. coli.*

	MDR		NonMDR		*p*-value	odds ratio	95% confidence interval
	Frequency	%	Frequency	%			
*Biofilm producer (n* = 73)	58	79.5%	15	20.5%	0.015	2.37	1.181–4.757
*Biofilm nonproducer (n* = 100)	62	62.0%	38	38.0%		1	
*Diabetic (n* = 61)	45	73.8%	16	26.2%	0.354	1.387	1.694–2.775
*Nondiabetic (n* = 112)	75	67.0%	37	33.0%		1	

## Data Availability

The data used to support the findings of this study are available from the corresponding author upon request.
